# Human induced pluripotent stem cell-derived cardiomyocytes as an electrophysiological model: Opportunities and challenges—The Hamburg perspective

**DOI:** 10.3389/fphys.2023.1132165

**Published:** 2023-02-16

**Authors:** Djemail Ismaili, Carl Schulz, András Horváth, Jussi T. Koivumäki, Delphine Mika, Arne Hansen, Thomas Eschenhagen, Torsten Christ

**Affiliations:** ^1^ Institute of Experimental Pharmacology and Toxicology, University Medical Center Hamburg-Eppendorf, Hamburg, Germany; ^2^ Department of Cardiology, University Heart and Vascular Center Hamburg, University Medical Center Hamburg-Eppendorf, Hamburg, Germany; ^3^ DZHK (German Centre for Cardiovascular Research), Partner Site Hamburg/Kiel/Lübeck, Hamburg, Germany; ^4^ Translational Cardiology, Department of Cardiology, Inselspital, University Hospital Bern, University of Bern, Bern, Switzerland; ^5^ BioMediTech, Faculty of Medicine and Health Technology, Tampere University, Tampere, Finland; ^6^ Inserm, UMR-S 1180, Université Paris-Saclay, Orsay, France

**Keywords:** hiPSC-CM, hESC-CM, human cardiomyocytes, ion currents, action potentials, computer modelling and simulation, automated patch clamp

## Abstract

Models based on human induced pluripotent stem cell-derived cardiomyocytes (hiPSC-CM) are proposed in almost any field of physiology and pharmacology. The development of human induced pluripotent stem cell-derived cardiomyocytes is expected to become a step forward to increase the translational power of cardiovascular research. Importantly they should allow to study genetic effects on an electrophysiological background close to the human situation. However, biological and methodological issues revealed when human induced pluripotent stem cell-derived cardiomyocytes were used in experimental electrophysiology. We will discuss some of the challenges that should be considered when human induced pluripotent stem cell-derived cardiomyocytes will be used as a physiological model.

## Introduction

The development of human induced pluripotent stem cell-derived cardiomyocytes (hiPSC-CM) is expected to become a relevant step for the translational aspect of cardiovascular basic research. HiPSC-CM provide human-based disease models for detailed functional studies of pathogenesis in a patient-specific manner. Importantly, hiPSC-CM-based models should allow to quantify the impact of genetic editing on an electrophysiological background close to the situation in humans. We share the enthusiasm on application of hiPSC-CM as a milestone in cardiovascular research. However, using hiPSC-CM in experimental electrophysiology during the past years, we have identified some challenges that we will discuss here and that are hopefully of interest to a broader readership.

## Ion currents in hiPSC-CM: All aboard or even stowaways?

**FIGURE 1:**
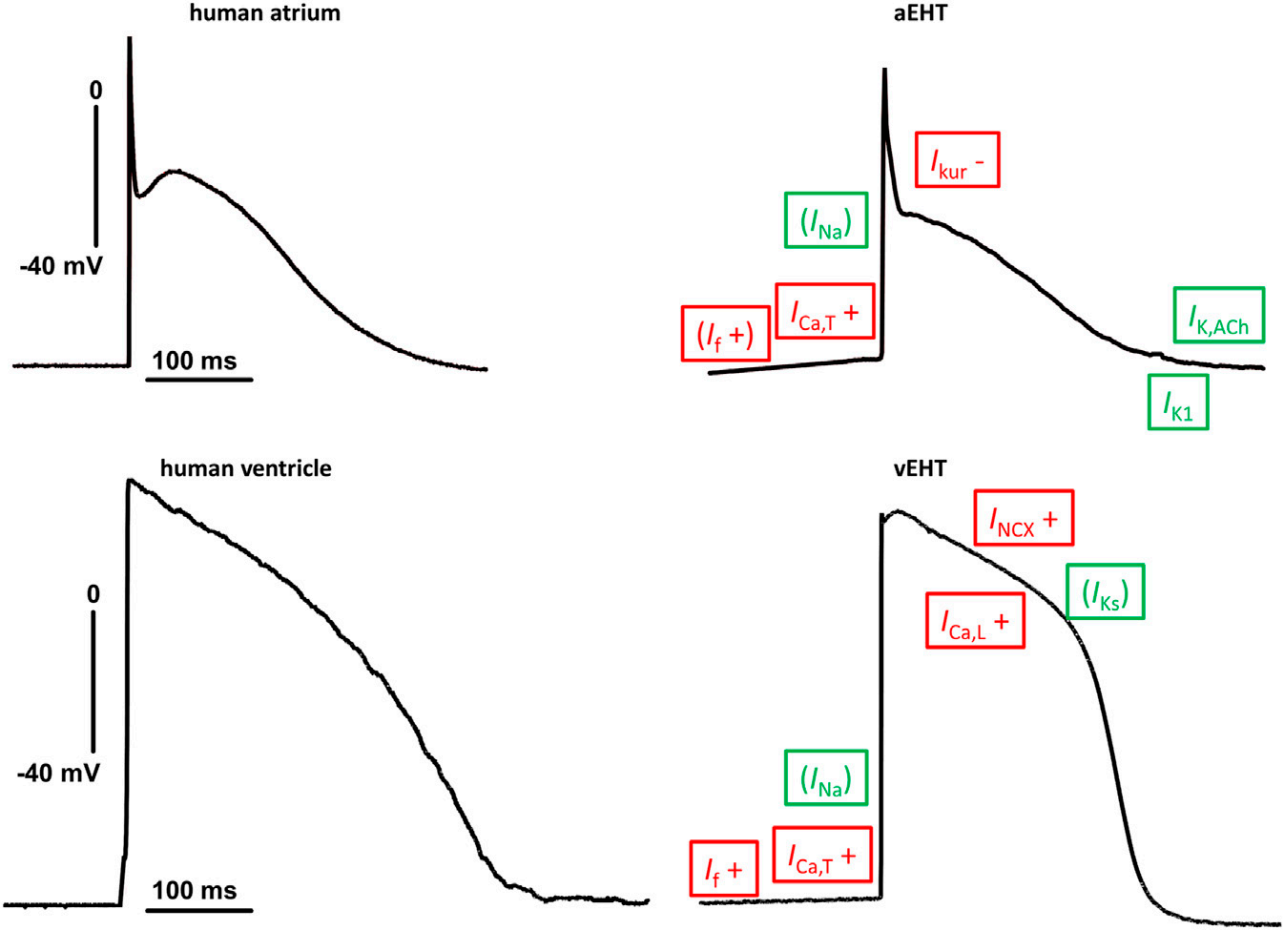
Similarities and differences in action potential shapes between adult human heart tissue and engineered heart tissue based on hiPSC-CM. Typical original traces of action potentials (AP) recorded in human adult (left) and engineered heart tissue (EHT, right) from atrium (top) and ventricle (bottom). Same scaling and zero voltage level for all four pictures. Differences in ion currents are indicated with red letter with + or − indicating changes related to adult tissue. Ion currents within the same range are indicated in green letter. Letters are given in brackets for ion currents where differences are suspected from AP recordings (no direct comparison of patch clamp data). Abbreviations: *I*_Ca,L_, L-type Ca^2+^-current; *I*_Ca,T_, T-Type Ca^2+^-current; *I*_f_, hyperpolarization-activated funny current; *I*_K,ACh_, Acetylcholine-activated potassium current; *I*_Ks_, slow delayed rectifier potassium current; *I*_Kur_, ultrarapidly activating potassium current; I_K1_, inward rectifier potassium current; *I*_Na_, sodium current; *I*_NCX_, Na^+^/Ca^2+^-exchanger.

Data for a large number of ion channels in hiPSC-CM were published in 2011 (Ma et al., 2011) and following results were compared in detail to data obtained in the human adult heart (Figure 1; Casini et al., 2017). Given the importance of pump and exchanger currents for the electrical stability of cardiomyocytes (CM), data on Na^+^/Ca^2+^-exchanger (NCX) and Na^+^/K^+^-ATPase (NKA) in heart muscle, data for hiPSC-CM were reported as a following step in 2012 (Fine et al., 2013). Curiously, no data on NCX currents in human ventricular CM were available at that time. Data in hiPSC-CM fit nicely to the situation in native human atrial CM, when NCX was measured under the same experimental conditions (Christ et al., 2016). A recent study, directly comparing NCX currents in hiPSC-CM (cultured in engineered heart tissue, EHT) and adult human ventricular CM, found the NCX current density to be slightly higher in hiPSC-CM (Ismaili et al., 2022). However, the functional relevance of NCX cannot be predicted from current densities alone, since robust NCX currents were found consistently in ventricular myocytes of various species but functional consequences of pharmacological NCX-block on action potential duration (APD) and contractile force differ widely [for discussion see (Ismaili et al., 2022)]. Block of NCX shortened APD at 90% repolarization (APD_90_) in EHT but not in human left ventricular tissue. In line with the APD data, force was increased by NCX-block in hiPSC-CM but not in human left ventricle (Ismaili et al., 2022). The lack of effect of NCX block on APD and force in human ventricular tissue is perplexing but fits well to earlier results in human atrial tissue (Christ et al., 2016). It should be noted that NCX block is devoid of effects on APD and force in many larger animals and we suppose that still more research is needed to better understand the physiological relevance of NCX in the adult heart (Eisner and Sipido, 2004). Inward currents by NCX resulting from Ca^2+^-release should contribute to pacemaking in hiPSC-CM (Morad and Zhang, 2017). Newly developed, highly selective blockers of NCX are available (Otsomaa et al., 2020). However, somewhat unexpectedly block of NCX has only marginally effects on beating rate in adult animal sinus node (Kohajda et al., 2020). Data on beating rate in hiPSC-CM are missing.

The situation regarding NKA is similarly suboptimal. The current densities for NKA in hiPSC-CM (Fine et al., 2013) fit nicely to data obtained in human atrial CM (Workman et al., 2003) but no data on NKA currents exist for human ventricular CM so far. As reported for human ventricular tissue (Prasad and Callaghan, 1970; Schwinger et al., 1990), inhibition of NKA by ouabain increased force in EHT (Mannhardt et al., 2016). Of note, ouabain exerted a biphasic inotropic response in EHT with an increase in force at low to medium (0.3–1 µM) and a sharp decrease at higher concentrations. In a study in human ventricular tissue, ouabain (200 nM) shortened APD_90_ (Prasad and Callaghan, 1970) and induced early afterdepolarizations (EAD) (Dangman et al., 1982). The n numbers are small and results were never confirmed. No data on effects of NKA-block on action potential (AP) are available for hiPSC-CM.

In the human ventricle, expression of transporters and ion channels show a transmural gradient (Soltysinska et al., 2009). Detailed biophysical characterization of potassium outward currents in human ventricular CM revealed an important role of the transient potassium outward current (*I*_to_) for the transmural gradient of repolarization (more in subepicardial than in subendocardial layer) (Wettwer et al., 1994). *I*_to_ was reported in several hiPSC-CM cell lines (Ma et al., 2011; Lemme et al., 2018; Flenner et al., 2021). However, there is concern that *I*_to_ contributes less to AP shape in hiPSC-CM. That is, high concentrations of the prototypical *I*_to_ blocker 4-aminopyridine (4-AP) failed to affect AP shape in two of our in-house control hiPSC-CM cell lines (Lemme et al., 2018; Schulz et al., 2023). This finding may substantially limit the value of hiPSC-CM as a disease model that involves changes in *I*_to_ (Flenner et al., 2021) and indicate that actual hiPSC-CM preparations may represent a subendocardial ventricular CM phenotype (Wettwer et al., 1994). Thus, it remains open whether the generation of hiPSC-CM with a more subepicardial AP shape is a realistic goal.

Consequently, there is legitimate concern whether hiPSC-CM express all cardiac ion channels and transporters and whether their relative contribution fits the situation in the human heart (Casini et al., 2017). Conversely, hiPSC-CM clearly express ion channels that are absent in the adult human working myocardium.

Spontaneous beating and the sensitivity of hiPSC-CM beating rate to ivabradine strongly suggest substantial activity of the hyperpolarization-activated funny current (*I*_f_) in hiPSC-CM EHT (Mannhardt et al., 2017). Accordingly, transcript abundance of HCN4 in hiPSC EHT is relatively high. In bulk RNA sequencing analysis, the mean of 28 control lines amounted to 3300 reads per million (=#807 most expressed gene; unpublished), while it is absent in normal human ventricles (Litviňuková et al., 2020). *I*_f_ density in standard ventricular-like hiPSC-CM was reported to be ∼5 pA/pF, i.e., in the same range of human sinus node cells (Giannetti et al., 2021), but also in specifically differentiated sino-atrial hiPSC-CM (Protze et al., 2016). Biophysical properties of *I*_f_ and its autonomic modulation are very close to the situation in rabbit sinus node cells, making hiPSC-CM an attractive model to study cardiac pacemaking (Giannetti et al., 2021). A detailed study in hiPSC-CM revealed biochemical and electrophysiological changes in HCN4 in Coxsackievirus B3 (CVB3)-infected hiPSC-CM, a phenomenon that may contribute to the clinically observed myocarditis-induced bradycardia and cardiac arrest (Peischard et al., 2022). The latter study is remarkable, since it nicely illustrates that successful application of hiPSC-CM is not necessarily restricted to genetic studies.

Another current absent in the human working myocardium but occasionally found in hiPSC-CM is the T-Type Ca^2+^-current (*I*_Ca,T_) (Ivashchenko et al., 2013; Uzun et al., 2016). The presence of *I*_Ca,T_ cannot necessarily be taken as an indication of immaturity, since this current has been found in the adult ventricular myocardium of many species (Uzun et al., 2016). The co-existence of T-type and L-type Ca^2+^-channels in hiPSC-CM suggests that the additional inward current at potentials negative to the activation threshold for L-type Ca^2+^-currents (*I*_Ca,L_) likely contributes to pacemaking in hiPSC-CM. Moreover, it has to be taken into account that a substantial fraction (20%–30%) of peak Ca^2+^-current can consist of *I*_Ca,T_ in hiPSC-CM subjected to a typical test pulse potential used to measure *I*_Ca,L_ (+10 mV) (Uzun et al., 2016). This finding can lead to overestimation of real Ca^2+^-influx, since *I*_Ca,T_ inactivates much faster than *I*_Ca,L_. Thus it may be wise to quantify *I*_Ca,L_ as dihydropyridine-sensitive currents in hiPSC-CM that possess *I*_Ca,T_ (Prondzynski et al., 2019). Taken together, hiPSC-CM can express cardiac ion channels such as *I*_f_ and *I*_Ca,T_ that are not expressed in adult human ventricular CM.

In addition, non-cardiac ion channels can be present in hiPSC-CM. An example are Big Conductance Calcium Activated Potassium Currents (*I*_BK,Ca_) that were shown to be responsible for induced afterdepolarizations and oscillations in EHT of a specific hiPSC line (Horváth et al., 2020). Identification of *I*_BK,Ca_ was rather easy because of a bizarre AP morphology and the availability of a selective blocker (iberiotoxin) (Horváth et al., 2020). Given that the current was not observed in EHTs from three other independent control lines and the transcript of the underlying channel (KCNMA1) was essentially absent in 44 other independent lines (unpublished data), a hiPSC artefact seems likely. Indeed, two independent reports demonstrated that iPSCs frequently acquire genetic alterations during cell culture. Kilpinen et al. (2017) showed that chromosome 10, harboring BKCa/KCNMA1, was among the most susceptible locus to copy number alterations. Another study searching for variants that provide mutated cells with a growth advantage in culture, identified KCNMA1 variants in two independent stem cell lines (Merkle et al., 2017). Thus, a genetic alteration leading to a reoccurring overgrowth of BK and misexpression in hiPSC is the most likely explanation for our finding. It remains open if this example may represent an exotic exception or if it is just the “tip of an iceberg.” Anyhow, expression of non-cardiac sarcolemmal ion channels should be considered.

## Action potentials recording in hiPSC-CM: Methodological issues

Initially, cultures of hiPSC-CM were interpreted as a mixture of ventricular, atrial and nodal CM. The interpretation was based on differences in resting membrane potential (RMP) and APD, which were analyzed in small numbers of cells (*n* ∼ 20–30) by patch clamping (Itzhaki et al., 2010; Moretti et al., 2010; Ma et al., 2011; Liang et al., 2013; Ma et al., 2013; Liang et al., 2016). However, analysis in a larger population (*n* = 320) of hiPSC-CM showed that the frequency distribution for APD_90_ followed a single Gaussian distribution, arguing against the idea of a truly mixed cell population and for a simple scatter of data (Du et al., 2015). Measurements were done by voltage-sensitive dyes that does not allow any conclusion on (diastolic!) membrane potential and it was argued that this limitation may have weakened the power to detect the mentioned above different populations (Giles and Noble, 2016). It is important to note that APD_90_ did not significantly differ between human right atrium and left ventricle (measured in intact tissue by sharp microelectrodes) (Horváth et al., 2018). In contrast, RMP was more negative in human left ventricle than in right atrium (measured by sharp microelectrodes) (Horváth et al., 2018). Nevertheless the difference was small (2.5 mV) and the groups showed substantial overlap (Horváth et al., 2018), making RMP less appropriate to distinguish between atrial and ventricular CM. The suitability of a parameter to distinguish between atrial and ventricular hiPSC-CM does not only depend on its power to discriminate between intact human adult atrial and ventricular tissue, but also on the method to measure action potentials. Most researchers have measured AP in isolated cells with patch electrodes. By using this approach, diastolic potentials are less negative in hiPSC-CM than in adult CM. Consequently, application of hyperpolarizing holding currents is mandatory in hiPSC-CM to bring the membrane potential in a range negative enough to elicit an AP. Clearly, such a “hold” diastolic membrane potential cannot be longer used as a discriminator between atrial and ventricular CM.

The less negative diastolic potential of hiPSC-CM has attracted much attention and is generally assumed a marker of immaturity of the CM and explained by abnormally low current densities of the inward rectifier current (*I*_K1_) (Ma et al., 2011). However, *I*_K1_ densities are not necessarily too low in hiPSC-CM (Horváth et al., 2018). In fact, we measured mean values of barium (1 mM)-sensitive inward rectifying currents of 33 pA/pF in hiPSC-CM, compared with 41 pA/pF in left ventricular CM. We suspect technical issues may contribute. HiPSC-CM are much smaller than adult CM (Uzun et al., 2016). Even if a hiPSC-CM possesses physiological inward rectifier density, total membrane conductivity is much lower than in adult CM. Thus, membrane resistance is high and is close to the range of seal resistance, which is expected to generate substantial voltage errors. Such voltage errors can be avoided when membrane potential is measured by sharp microelectrodes in intact tissue. This approach became available with the introduction of EHT and was reported as early as in 2002 (Eschenhagen et al., 2002).

There are other advantages of sharp microelectrodes to measure AP. Proper cell-to-cell coupling may be critical for the generation of a correct diastolic potential in hiPSC-CM (Van de Sande et al., 2021). From a more general perspective, variability of APD is much lower when measured in intact tissue than in isolated CM measurements, because of high inter-cell variation of ion channel activity (Lachaud et al., 2022). This is not specific for hiPSC-CM, because the coefficient of variation was 2-fold and 3-fold higher for APD_90_ measured in isolated CM from human ventricle and atrium, respectively, than in intact tissue. Similarly, the variability was 5 times larger in hiPSC-CM than in EHT (Horváth et al., 2018), underscoring that AP recordings in isolated hiPSC-CM are particularly prone to methodological issues (Eschenhagen et al., 2002).

AP recordings in EHT also allow much longer lasting experiments (hours vs. minutes in case of hiPSC-CM) and thus application of several concentrations of a single test compound or application of different compounds (Lemoine et al., 2018) as routinely done in cardiac safety pharmacology on animal heart tissue (Himmel et al., 2012).

## Atrial models: How much atrial-like is enough?

There is great interest in hiPSC-CM for studying atrial electrophysiology in the context of atrial fibrillation (AF). Strong associations are known between gene variants and the risk to develop AF. Atrial hiPSC-CM should be the model of choice to measure the impact of a distinct genetic background on human atrial electrophysiology. The Passier and Keller groups have shown that addition of retinoic acid (RA) in the differentiation protocol from hiPSC to CM suffices to induce atrial differentiation in stem cell derived CM (Lee et al., 2017). Here we will focus on RA´s ability to induce a true atrial-like AP shape in hiPSC-CM. Additionally, CRISPR/Cas9-based targeting of fluorescent reporters has been used to target ventricular hiPSC-CM (expressing myosin light chain 2) (Luo et al., 2021) or atrial hiPSC-CM (expressing sarcolipin) (Chirikian et al., 2021). In the absence of RA the percent of atrial cells was low (between 0.6% and 2.7%, depending on the culture duration). Cells identified as atrial cells showed an effect upon low concentrations (100 µM) of 4-AP. However, they missed the typical spike and dome shape and 4-AP prolonged but did not shortened APD_90_ (Wettwer et al., 2004), indicating a rather incomplete atrial AP phenotype (Schulz et al., 2023).

In order to estimate the efficacy of RA to induce an atrial-like AP shape, parameters are needed that allow clear discrimination between ventricular and atrial tissue. In contrast to APD_90_ and RMP (discussed above), the repolarization fraction (RF) is a powerful discriminator with human atrial tissue showing high and ventricular tissue low RF (Horváth et al., 2018). RF was uniformly low in hiPSC-CM/EHT not treated with RA (Itzhaki et al., 2010; Merkle et al., 2017), indicating a predominantly ventricular phenotype. RF was substantially higher in hiPSC-CM treated with RA, indicating a more atrial phenotype (Lemme et al., 2018). The high RF in atrium reflects stronger initial repolarization and relates to larger transient outward currents than in the human ventricle (Amos et al., 1996). Transient outward currents not only differ quantitatively between atrium and ventricle, but also qualitatively. The ultrarapidly activating potassium current (*I*_Kur_) contributes to repolarization in the human atrium but not in the ventricle (Ravens et al., 2013). Pharmacological block of *I*_Kur_ by low concentrations of 4-AP (10–100 µM) allows detection of *I*_Kur_ in voltage clamp experiments (Amos et al., 1996). Furthermore, 4-AP enables to estimate contribution of *I*_Kur_ to AP shape, since a much higher concentration (millimolar range) is needed to block the transient outward current (present also in human ventricle). Thus, the sensitivity of the AP shape to low concentrations of 4-AP safely differentiates between the two regions. Finally, acetylcholine activates the atrial-selective acetylcholine-activated potassium current (*I*_K,ACh_), shorten APD_90_ and hyperpolarize the RMP in atrial, but not in ventricular tissue (Lemme et al., 2018). Of note, acetylcholine antagonizes effects of cAMP-dependent drugs such as isoprenaline in both tissues, the well-known indirect antiadrenergic effect (Méry et al., 1997).

In the seminal paper that showed the efficacy of RA to induce atrial differentiation in human embryonic stem cell (hESC)-derived CM, currents sensitive to 4-AP (50 µM) accounted to ∼6 pA/pF (pulse potential of +50 mV) (Devalla et al., 2015), which is 2-fold lower than values for *I*_Kur_ in human atrial CM (Ford et al., 2013). A later study in hiPSC-CM showed even smaller currents (∼2 pA/pF) (Hilderink et al., 2020), but they were sufficient to produce a short APD at 20% repolarization (APD_20_; 20 ms), close to the situation in human atrium (7 ms) (Liang et al., 2016; Horváth et al., 2018). Furthermore, as in human atrium, plateau voltage was below 0 mV, and both short APD_20_ and negative plateau voltage were sensitive to 50 µM 4-AP (Devalla et al., 2015). However, the effects of *I*_Kur_ block on final repolarization differed. While in atrial hESC-CM block of *I*_Kur_ (with 4-AP or by knockout of K_V_1.5) clearly prolonged APD_90_ (Devalla et al., 2015; Marczenke et al., 2017), it shortened APD_90_ in human atrium (4-AP and several newly developed *I*_Kur_ blockers) (Wettwer et al., 2004; Christ et al., 2008; Ford et al., 2013; Ford et al., 2016). This discrepancy, which is due to an indirect stimulation of *I*_Kr_ by the more positive plateau current (Wettwer et al., 2004), may have relevant consequences for the application of hiPSC-CM as a model for atrial drug screening. In fact, pharmacological blockers of *I*_Kur_ have gained great interest since they were believed to effectively terminate AF without the risk of life-threatening ventricular proarrhythmic effects. However, the inefficacy of *I*_Kur_ block to prolong atrial refractoriness *in vitro* (Du et al., 2015; Lemoine et al., 2018; Van de Sande et al., 2021; Lachaud et al., 2022) may explain the lack of antiarrhythmic efficacy in AF in patients (Shunmugam et al., 2018; Camm et al., 2019). Therefore, any pharmacological model where *I*_Kur_ block increases APD_90_ could be misleading (Devalla et al., 2015).

HiPSC-CM can be used to study tachypacing-induced remodeling in ventricular but also in atrial hiPSC-CM. In atrial tissues of patients with AF, *I*_Kur_ is smaller (Van Wagoner et al., 1997) and APD_20_ longer, resulting in a more triangulated (lower RF) AP. Importantly, APD_90_ is no longer shortened by 4-AP (Wettwer et al., 2004). One of our in-house RA-treated hiPSC-CM cell line shows some typical atrial characteristics (effect of 4-AP on APD), but the long APD_20_ resembles more the situation in chronic human AF (30 vs. 29 ms) (Ravens et al., 2015; Lemme et al., 2018). This finding could explain why we failed to induce remodeling in tachypaced atrial EHT (Lemoine et al., 2021).

Basal inward rectifier currents are smaller in human atrium than in ventricle (Varró et al., 1993; Horváth et al., 2018). There are no studies that compared basal inward rectifier currents between stem cell-derived CM treated with RA or not, but in small datasets activation of the *I*_K,ACh_ was restricted to RA-treated stem cell-derived CM (Devalla et al., 2015; Lee et al., 2017; Argenziano et al., 2018). In one study, the effect of muscarinic receptor activation on APD_90_ in RA-treated atrial hESC-CM was hard to detect at all (Devalla et al., 2015), but accounted to ∼20%–30% in atrial hiPSC-CM or atrial EHT (Lemme et al., 2018; Goldfracht et al., 2020), close to the situation in human atrium (Dobrev et al., 2001). *I*_K,ACh_ is another atrial-selective potassium current that undergoes electrical remodeling. Shortening of APD_90_ upon activation of *I*_K,ACh_ is lost in AF and constitutively active *I*_K,ACh_ (Dobrev et al., 2005) is believed to contribute to increased inward rectifier in AF (Van Wagoner et al., 1997). Therefore, it is obvious that a substantial *I*_K,ACh_ under control condition is necessary to study impact of tachycardic remodeling on inward rectifier currents.

In the human (but not rodent) heart, serotonin increases *I*_Ca,L_ and force in the atrium but not in the ventricle (Jahnel et al., 1992; Pau et al., 2003). The physiological relevance still remains open. Under pathological conditions such as chronic AF, serotonin responses undergo complex remodeling: positive inotropic and proarrhythmic effects are diminished (Pau et al., 2007; Christ et al., 2014), while the increase in *I*_Ca,L_ remains intact (Berk et al., 2016), underlining potential interest to use serotonin as a marker for AF-induced remodeling. It is not known whether atrial hiPSC-CM show an increase in *I*_Ca,L_ and force in response to serotonin.

## How specific and robust are computational models in recapitulating the hiPSC-CM phenotype(s)?

Biophysics-based computational modelling and simulations are a useful tool for quantitative characterization and understanding of the highly variable hiPSC-CM phenotype. To our knowledge, two different comprehensive approaches have thus far been employed in this context that we will refer to, for the sake of brevity, as data-based and population-based. Both approaches build a collection of virtual hiPSC-CMs by varying the model parameter values.

In the data-based approach that was first applied by Kernik et al. (2019), the parameter value ranges are derived directly from *in vitro* experiments. Whereas, in the population-based approach, which was introduced to hiPSC-CM realm by Paci et al. (2017), a chosen set of model parameter values are first varied in arbitrarily defined ranges, focusing on the maximum conductances and rates of ion channels and transporters. Then, the obtained virtual hiPSC-CM population is pruned by comparing a defined set of functional biomarkers to *in vitro* measurements. The fundamental limitation of the population-based approach is that despite the pruning the final collection will contain virtual hiPSC-CMs, whose parameter combinations are unphysiological in that they do not necessarily exist *in vitro*. The situation is analogous to using AP morphology as a marker for chamber specificity, because a ventricular- or atrial-like AP shapes can potentially be a result of underlying ion current strength combinations that do not exist in native human CMs. The data-based approach, on the other hand, is limited, well, by the available data. Despite the progress of high-throughput methods, measuring everything in one lab is not realistic. Thus, when the dataset is expanded to include measurements from multiple labs, the physiological variability will be exacerbated by inter-lab variability. More importantly, due to practical reasons, the depth of phenotype characterization in this approach will be limited, as it was in the Kernik et al. (2019)s study. Therefore, the model formulations need to be biophysically less detailed with fewer parameters so that their values can actually be measured *in vitro*. Thus, the model’s application area is restricted to the phenotypic space, which the data in question present—limited robustness. On the other hand, the more detailed hiPSC-CM models that have been employed in the population-based approach follow the common iterative modelling tradition, inheriting some model components and their parameters from previous, non-hiPSC-based, model generations—limited specificity.

Despite the limitations mentioned above, and others not discussed here, computational modelling is perhaps the only way to make sense of *in vitro* hiPSC-CM data, quantitatively. Although there are substantial and continuous methodology development efforts to narrowing the gap, the hiPSC-CM phenotype is different from native human CMs; a fact with which the cardiac research community has luckily come to terms with. The capabilities of the so-called computational maturation in translating the hiPSC-CM-based findings to the human context have already been shown in multiple independent studies (Gong and Sobie, 2018; Koivumäki et al., 2018; Tveito, Jæger, Huebsch, Charrez, Edwards, Wall, Healy; Jæger et al., 2020). Wider adoption of such frameworks would probably be fostered by transparent comparisons of the cell models as well as the inversion and regression approaches used in translation, as done recently for example, by Paci et al. (2021).

## HiPSC-CM and automated patch clamp: The dream wedding in experimental cardiac electrophysiology?

The advent of hiPSC-CM coincides with the development of automated patch clamp (APC). The technique allows high-throughput experiments and has therefore successfully replaced the technically demanding and time-consuming conventional patch clamp in the field of drug screening in cellular expression systems. Besides the much higher throughput, APC offers the unique possibility to exchange the intracellular solution during an experiment, a procedure that is hard to achieve in manual patch clamp. In principle, APC in hiPSC-CM could be able to combine the benefits of hiPSC-CM (human background, genetically defined or even edited systems) with high-throughput measurements. Until very recently, APC had failed in large, elongated adult CM. In contrast, isolated ball-shaped hiPSC-CM resemble the cells (e.g., CHO, HEK) of commonly used heterologous expression systems and could therefore be better suited for APC. In fact, sodium current (*I*_Na_) and *I*_Ca,L_ are large in any type of CMs and can therefore routinely be measured by APC in hiPSC-CM (Goversen et al., 2018; Li et al., 2019). Ion current measurements by APC in hiPSC-CM bearing patient mutations could be of great interest and one recent study used hiPSC-CM derived from patients with Brugada-syndrome AP (Li et al., 2021). In contrast, we are not sure whether ion current measurements in hiPSC-CM from healthy donors will replace “traditional” measurements in expression system (Lemoine and Christ, 2021). The main reason lies in the fact that, even in human ventricular myocytes, repolarizing potassium outward currents are very small and difficult to detect. Delayed rectifier K^+^ channel currents in adult human cardiac myocytes amount to less than 1 pA/pF (Jost et al., 2013) or remained undetectable [for detailed discussion compare (Ma et al., 2011)]. Moretti et al. (2010) were the first to demonstrate slow delayed rectifier currents (*I*_Ks_) in hiPSC-CM. Ma et al. (2011) showed biophysical characteristics of delayed rectifier in hiPSC-CM close to the situation in humans. Interestingly, the authors found *I*_Ks_ currents in only 50% of hiPSC-CM, as reported earlier for human ventricular CM (Virág et al., 2001). Even with manual patch clamp only few studies report delayed currents (both *I*_Ks_ and *I*_Kr_) in hiPSC-CM (Ma et al., 2013; Zhang et al., 2014). There is concern whether biophysical properties of *I*_Kr_ in hiPSC-CM resemble the situation in human CM (Zhang et al., 2014). It is unclear whether the issue relates to biological or methodological aspects (Christ et al., 2015). At present we are not aware of data on delayed rectifier currents in hiPSC-CM obtained by APC.

The shape of the AP represents a sum parameter of all ion channels active in a relatively intact cell/tissue. This may explain why (drug) effects on repolarization can be easily detected, making AP recording by APC highly attractive (Scheel et al., 2014). From the beginning, APD was consistently shorter in isolated hiPSC-CM when measured by APC than APD measured in intact adult human ventricular tissue by conventional sharp microelectrodes (Friedrichs et al., 2015). Holding currents (see above), essential to bring the diastolic potential in a range that allows Na^+^-channel activation, could be responsible for the short APD. The issue with too small inward rectifier in hiPSC-CM could be fixed by dynamic clamping (Verkerk et al., 2017; Verkerk AO and Wilders, 2020). However, individual adjustment of dynamic clamp current density is limited to cell capacitance (Becker et al., 2020) but not to actual I_K1_ even in advanced APC systems. High membrane resistance of small cells like hiPSC-CM promotes voltage errors. The issue is of particular importance in case of improper seal resistances. Only one study using APC in ventricular-like hiPSC-CM reported some indirect information about seal resistance. The authors used a seal resistance >100 MΩ as a criterion for successful patching (Seibertz et al., 2022). Actual values in the data set were not reported. However, we would suspect that much higher values would be necessary to record the true membrane potential, values that are difficult to achieve even with manual patch clamp. In the same study authors had to apply continuous holding currents (mean value of −9.7 ± 1.5 pA/pF) to allow upstroke generation (Seibertz et al., 2022). Such holding currents are almost fivefold higher than peak I_Kr_ current in hiPSC-CM (Feng et al., 2021) and may at least in part explain the very short APD_50_ reported in this study [∼70 ms, which compares with 220 ms in adult human ventricular tissue (Skibsbye et al., 2014)].

Thus, the high throughput in APC comes at a price. It is clearly an advantage if large numbers of compounds are to be tested during drug development. It also opens the possibility to phenotype hiPSC-CM from cell lines of larger patient populations. However, given the above considerations, such approaches are presently restricted to robustly measurable currents such as *I*_Na_ and *I*_Ca,L_ (Seibertz et al., 2022). The log-fold increase in number of experiments will be helpful to cope with data scattering, a phenomenon whose basis we are just beginning to better understand (Ismaili et al., 2020). We are sceptical if APC is useful for AP recordings in hiPSC-CM. However, APC could help to increase the power of discrimination when ion currents are measured that differ only slightly between experimental groups.

## Regulation of ion currents by cAMP

Ca^2+^-handling in heart muscle is under strong control of the sympathetic nervous system. One of the many effectors, *I*_Ca,L_, can be easily monitored by patch clamp. Regulation of *I*_Ca,L_ by cAMP/PKA pathway is described in high detail in adult CM from many mammalian species including humans (Liu et al., 2020; Hovey et al., 2022; Papa et al., 2022). Thus, measurements of *I*_Ca,L_ in hiPSC-CM should help to elucidate how closely regulation resembles the situation in human adult myocardium.

As in the human heart, *I*_Ca,L_ in hiPSC-EHT can be robustly increased by activation of both β_1_-and β_2_-adrenoceptors (Molenaar et al., 2014; Uzun et al., 2016). The potency of both epi- and norepinephrine to increase *I*_Ca,L_ was close to the situation in human CM. *I*_Ca,L_ increases were drastically smaller in hiPSC-CM cultured as monolayers than in EHT, while maximum cAMP increases (measured by Foerster energy transfer, FRET) were only slightly smaller (Uzun et al., 2016). Importantly, inhibition of PDE4 could bring increases in cAMP and *I*_Ca,L_ values in monolayers to values measured in EHT, indicating that PDE4 strongly restricts cAMP and its coupling to Ca^2+^-channels in hiPSC-CM cultured in monolayer format (Iqbal et al., 2021). Inhibition of PDE4 also potentiated inotropic responses to adrenergic stimulation in EHT, while inhibition of PDE3 did not (Saleem et al., 2020). The dominant impact of PDE4 seems to be a peculiarity of hiPSC-CM even in EHT format and contrasts with adult human myocardium, where PDE3 is the major PDE isoform restricting adrenergic responses (Eschenhagen, 2013; Molenaar et al., 2013).

As discussed above, *I*_f_ is consistently expressed in many hiPSC-CM cell lines and provides an opportunity to study regulation of ion currents by cAMP in hiPSC-CM. As in animal sinus node cells, *I*_f_ was increased by stimulation of β-adrenoceptors (by isoprenaline) but inhibited by stimulation of muscarinic receptors (by carbachol). Importantly, the potency of both compounds was close to values reported for rabbit sinus node cells (no data available from humans) (Giannetti et al., 2021), demonstrating that cAMP/PKA-dependent signaling is well developed in many hiPSC-CM.

## Outlook

We discussed some aspects of electrophysiological measurements in hiPSC-CM and the substantial progress in electrophysiological characterization of hiPSC-CM. However, results still vary widely between different studies. Reasons are expected in methodological issues in the recording techniques, but also in biological differences between cell lines and culture techniques (e.g., monolayers vs. EHT, age of cultures, different culture media). The multitude of differences implies that the comparability between studies is more challenging in hiPSC-CM than in traditional studies on animal or human heart tissues. Experimental electrophysiologists can help to reduce scatter by critical application and interpretation of the various recording methods. The enthusiasm about application of hiPSC-CM in experimental cardiac electrophysiology seems unbroken. Thus, we expect that the concerted expertise of experimental electrophysiologists and stem cell experts will solve the challenges step by step.

## Abbreviations

AP, action potential; APC, automated patch clamp; APD, action potential duration; APD_20_, APD at 20% repolarization; APD_90_, APD at 90% repolarization; CM, cardiomyocytes; EAD, early afterdepolarizations; EHT, engineered heart tissue; hESC-CM, human embryonic stem cell-derived cardiomyocytes; hiPSC-CM, human induced pluripotent stem cell-derived cardiomyocytes; *I*_BK,Ca_, Big Conductance Calcium Activated Potassium Current; *I*_Ca,L_, L-type Ca^2+^-current; *I*_Ca,T_, T-Type Ca^2+^-current; *I*_f_, hyperpolarization-activated funny current; *I*_K,ACh_, Acetylcholine-activated potassium current; *I*_Ks_, slow delayed rectifier potassium current; *I*_Kr_, rapid delayed rectifier potassium current; *I*_Kur_, ultrarapidly activating potassium current; I_K1_, inward rectifier potassium current; *I*_Na_, sodium current; *I*_to_, transient potassium outward current; NCX, Na^+^/Ca^2+^-exchanger; NKA, Na^+^/K^+^-ATPase; RA, Retinoic acid; RMP, resting membrane potential; RF, repolarization fraction; 4-AP, 4-aminopyridine.
